# Successful Nerve Block Therapy for Lateral Cutaneous Nerve Entrapment Syndrome (LACNES) Triggered by Exercise

**DOI:** 10.7759/cureus.36151

**Published:** 2023-03-14

**Authors:** Kumiko Yamada, Keiichi Shimazaki, Shinichi Inomata, Yohei Owada

**Affiliations:** 1 Department of Anesthesiology, University of Tsukuba Hospital, Tsukuba, JPN; 2 Division of Clinical Medicine, Department of Anesthesiology, Faculty of Medicine, University of Tsukuba, Tsukuba, JPN; 3 Department of Gastrointestinal and Hepato-Biliary-Pancreatic Surgery, Faculty of Medicine, University of Tsukuba, Tsukuba, JPN

**Keywords:** pulsed radiofrequency treatment, chronic abdominal wall pain, trigger-point injection, lateral cutaneous nerve entrapment syndrome, flank pain

## Abstract

This report presents a case of a 59-year-old man who experienced pain in the left abdomen during abdominal exercises, which gradually improved. Pain recurred in the same area one year later and gradually worsened, rendering him unable to work. The strongest tender point, with a positive Carnett's sign, was noted on the flank. Ultrasonography revealed a 5 × 10 mm mass shadow in the internal oblique muscle. Trigger point injection at the same site was remarkably effective. Lateral cutaneous nerve entrapment syndrome caused by a crush injury due to abdominal exercises was diagnosed. Nerve block therapy provided effective pain relief.

## Introduction

Abdominal wall pain is an overlooked cause of chronic undiagnosed abdominal pain. The prevalence of abdominal wall pain is 3% in emergency rooms with any type of chronic abdominal pain [1].

The prevalence of abdominal wall pain can be as high as 30% in patients whose previous diagnostic evaluation (usually intra-abdominal disease) was inconclusive [2,3]. Pain is usually caused by the entrapment of the cutaneous branches of sensory nerves that supply the abdominal wall. Anterior cutaneous nerve entrapment syndrome (ACNES) is a well-known cause of chronic abdominal wall pain. This is because the anterior cutaneous branch, a branch of the intercostal nerve, is compressed at the site penetrating the rectus abdominis muscle, causing abdominal wall pain in the lower abdomen [4].

The intercostal nerve has lateral and posterior cutaneous branches in addition to the anterior cutaneous branch, and the strangulation of these branches has recently been identified to cause neuropathic pain similar to that associated with ACNES [5]. Lateral abdominal pain, which is characteristic of neuropathic pain, may be due to strangulation of the lateral cutaneous branch of the intercostal nerve (lateral cutaneous nerve entrapment syndrome (LACNES)) [6,7]. The lateral cutaneous branch of the intercostal nerve extends anteriorly and posteriorly through the internal and external oblique muscles of the flank. Nerve strangulation can purportedly occur at the site that penetrates this muscle.

The signs and symptoms of ACNES and LACNES are similar. LACNES is a variant of the well-established ACNES and a form of underdiagnosed flank pain.

Herein, we report a case of successful nerve block therapy for LACNES triggered by abdominal muscle exercise.

## Case presentation

This is a case of a 59-year-old man with a height of 170 cm and a weight of 76 kg. His medical history was unremarkable, and he had a history of smoking 15 cigarettes per day for the past 30 years.

He noticed severe pain in the left flank while performing abdominal muscle exercises that comprised twisting the trunk to train the abdominal oblique muscles, which was spontaneously relieved. Subsequently, our inquiries revealed that he had no habit of exercising, but suddenly wished to develop his abdominal muscles and that he had performed abdominal muscle exercises for the first time in a long time. Approximately one year after the episode, in January, left abdominal pain at the same site appeared and gradually worsened, hindering his work. He visited a gastroenterologist and underwent contrast-enhanced CT, abdominal ultrasound, and blood sampling. Gastroenteral, urological, or cardiovascular diseases were not suspected. The findings were not suggestive of the cause of the flank pain. The patient was referred to pain medicine, as the etiology could not be determined.

**Figure 1:**
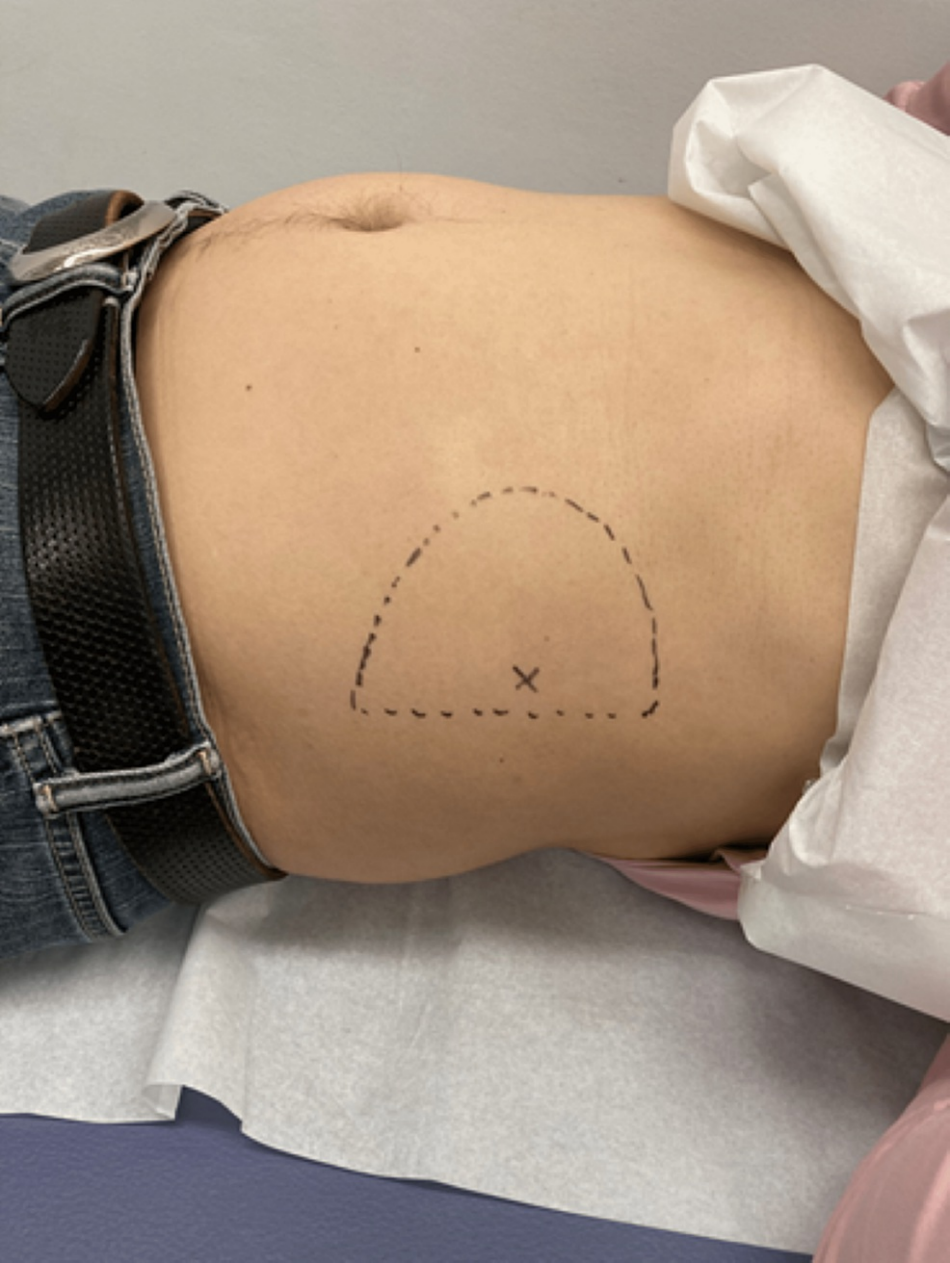
The site of abdominal pain The strongest pain point is indicated by (×), and the range of the surrounding pain is indicated by a dotted line. Hyperesthesia was observed along the dotted line.

At the first visit, his numerical rating scale (NRS: 0-10) score was 9. The pain worsened in the standing or sitting position. The pain severely restricted his daily activities. The strongest tender point with a positive Carnett's sign was noted at the intersection of the caudal side of the 11th costal cartilage and the axillary midline. The strongest pain point is indicated by (×), and the range of the surrounding pain is indicated by a dotted line in Figure 1. Hyperesthesia was observed along the dotted line.

**Figure 2:**
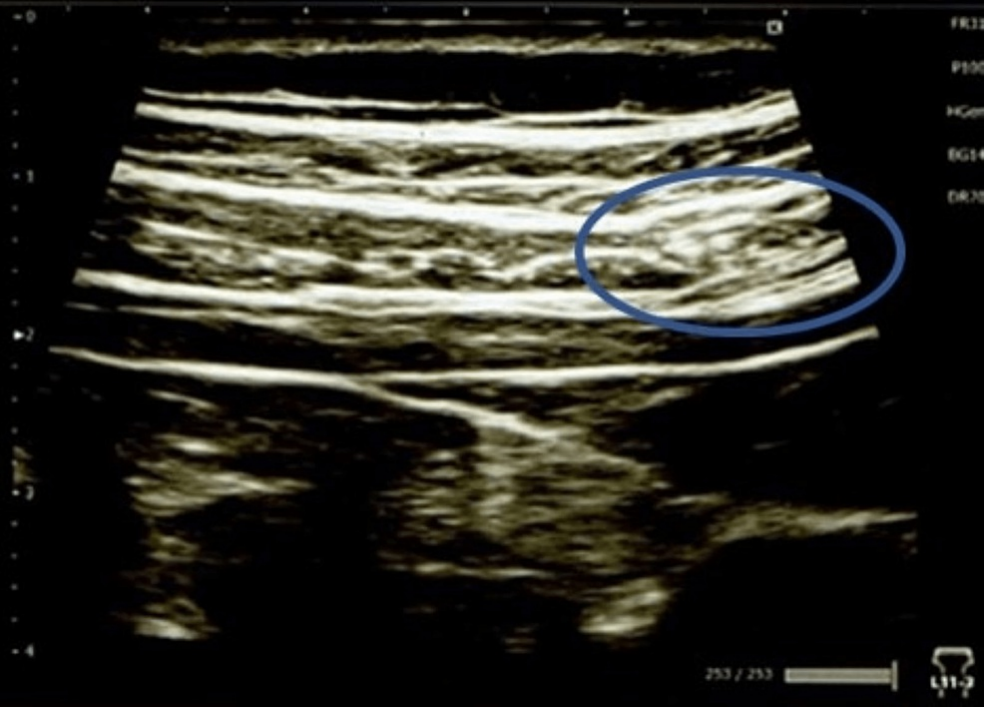
Ultrasonographic findings on the first visit A 5 × 10 mm mass shadow was observed in the internal oblique muscle, which coincided with the strongest pain point.

A 5 × 10 mm mass shadow was observed in the internal oblique muscle, which coincided with the strongest pain point (Figure 2).

An immediate effect was exhibited when 5 mL of sodium salicylate/dibucaine was administered around the mass shadow observed on ultrasonography. A repeat trigger point injection was administered when the pain returned two weeks later, and this allowed the patient to return to work. After the third trigger point injection, the pain at the strongest pain point subsided.

Mirogabalin was administered at 10 mg/day (5 mg twice daily; once in the morning and at night) because the surrounding pain remained.

Subsequently, the symptoms on the left side improved from an NRS score of 2 to 3. The symptoms of the strongest tender point were originally unclear, and a more central block was considered. An ultrasound-guided intercostal nerve block at the left Th11 level with 3 mL 0.375% ropivacaine was effective for the left flank pain. Since the effect was observed with the intercostal nerve block using a local anesthetic, a left Th11 intercostal nerve block using pulsed radiofrequency (PRF) was performed. It was performed for 300 seconds in PRF mode, and since then, the pain has changed from an NRS score of 2 to 3 without taking oral medication.

## Discussion

In this case, muscle injury might have occurred due to abdominal muscle exercise, and the intercostal nerves were involved in the healing process, resulting in neuropathic pain.

ACNES causes abdominal wall pain. ACNES occurs when the anterior cutaneous branch of the intercostal nerve, which runs between the internal oblique and lateral abdominal muscles, is compressed in the fibrous tissue ring within the rectus abdominis muscle.

The thoracic intercostal nerve is divided into three branches: anterior, lateral, and posterior. Spontaneous neuropathy flank pain may be due to the compression of the lateral branch of the thoracic intercostal nerve, which passes through the external intercostal and serratus anterior muscles toward the flank. While piercing these muscles, they divide into anterior and posterior branches. This site is probably where the nerves can be strangled, leading to typical neuropathic flank pain that suggests creating LACNES [6,7].

In a report examining ACNES, the onset factors were idiopathic (57%), recent abdominal surgery history (28%), accidents, sports injuries, and pregnancy [1]. Differential diagnoses included abdominal wall scar hernia, spinal disease, diabetes, and drug-induced neuropathy.

In our case, the patient’s medical history strongly suggested that abdominal exercise might have been a trigger.

The diagnostic criteria for LACNES include the following. Patients were eligible if at least three of the following four criteria were met: (1) a more than three months history of locoregional flank pain; (2) a constant area of tenderness located in the flank, covering a small fingertip point of maximal pain at the mid-axillary line; (3) a larger area of altered skin sensation, such as hypoesthesia, hyperesthesia, or altered cool perception, covering this maximal pain point, but not necessarily corresponding to a specific complete dermatome; (4) a positive pinch test (using thumb and index finger to “pinch” and lift the skin around the tender point eliciting a painful response in comparison to the contralateral side) [6].

Although the pinch test could not be performed in this case, the other criteria were met, strongly suggesting LACNES.

For diagnosis, abdominal ultrasound can be useful as a first-line investigation. In our case, the responsible lesion could not be identified by CT but was identified by ultrasound.

In 30 patients with suspected LACNES, all patients received trigger point injections. Of the patients, 83% reported more than 50% pain relief with the first injection [6].

After the first trigger point injection, repeated trigger point injections are effective in approximately half of the patients, and the other half reportedly require surgery, oral treatment, and pulse high-frequency method [6].

Like previous reports, multiple trigger point injections improved the symptoms of the strongest tender point in this case. The pain around the strongest point was difficult to resolve with trigger point injections in the present case. However, long-term pain relief could be achieved by blocking the intercostal nerves at the level of responsiveness to PRF.

## Conclusions

In our patient, trigger point injection and intercostal nerve block using PRF were successful for LACNES triggered by abdominal muscle exercise. ACNES, which is gaining increasing recognition, is a cause of abdominal wall pain. If symptoms similar to those of ACNES are observed on the side walls and back, the intercostal nerves may be entrapped and cause neuropathy pain, as in ACNES, and treatment at a pain clinic department with interventional pain techniques may be successful. In this case, by using the intercostal nerve block with the trigger point injection, we obtained an optimal effect on a wider range of pain.
